# Kidney Dyads: Caregiver Burden and Relationship Strain Among Partners of Dialysis and Transplant Patients

**DOI:** 10.1097/TXD.0000000000000998

**Published:** 2020-06-08

**Authors:** Sarah E. Van Pilsum Rasmussen, Ann Eno, Mary G. Bowring, Romi Lifshitz, Jacqueline M. Garonzik-Wang, Fawaz Al Ammary, Daniel C. Brennan, Allan B. Massie, Dorry L. Segev, Macey L. Henderson

**Affiliations:** 1 Department of Surgery, Johns Hopkins University School of Medicine, Baltimore, MD.; 2 McMaster University, Hamilton, ON.; 3 Department of Medicine, Johns Hopkins University School of Medicine, Baltimore, MD.; 4 Department of Epidemiology, Johns Hopkins School of Public Health, Baltimore, MD.; 5 Department of Acute and Chronic Care, Johns Hopkins University School of Nursing, Baltimore, MD.

## Abstract

**Background.:**

Caring for dialysis patients is difficult, and this burden often falls on a spouse or cohabiting partner (henceforth referred to as caregiver-partners). At the same time, these caregiver-partners often come forward as potential living kidney donors for their loved ones who are on dialysis (henceforth referred to as patient-partners). Caregiver-partners may experience tangible benefits to their well-being when their patient-partner undergoes transplantation, yet this is seldom formally considered when evaluating caregiver-partners as potential donors.

**Methods.:**

To quantify these potential benefits, we surveyed caregiver-partners of dialysis patients and kidney transplant (KT) recipients (N = 99) at KT evaluation or post-KT. Using validated tools, we assessed relationship satisfaction and caregiver burden before or after their patient-partner’s dialysis initiation and before or after their patient-partner’s KT.

**Results.:**

Caregiver-partners reported increases in specific measures of caregiver burden (*P* = 0.03) and stress (*P* = 0.01) and decreases in social life (*P* = 0.02) and sexual relations (*P* < 0.01) after their patient-partner initiated dialysis. However, after their patient-partner underwent KT, caregiver-partners reported improvements in specific measures of caregiver burden (*P* = 0.03), personal time (*P* < 0.01), social life (*P* = 0.01), stress (*P* = 0.02), sexual relations (*P* < 0.01), and overall quality of life (*P* = 0.03). These improvements were of sufficient impact that caregiver-partners reported similar levels of caregiver burden after their patient-partner’s KT as before their patient-partner initiated dialysis (*P* = 0.3).

**Conclusions.:**

These benefits in caregiver burden and relationship quality support special consideration for spouses and partners in risk-assessment of potential kidney donors, particularly those with risk profiles slightly exceeding center thresholds.

## Abstract 2

Supplemental Digital Content is available in the text.

## INTRODUCTION

Caring for dialysis patients is a difficult task and responsibility. Among spouses and cohabiting partners (henceforth referred to as dyads), end-stage renal disease (ESRD) requiring dialysis in 1 partner can decrease the quality of life (QOL) of both members of the dyad.^1–6^ Caregiving burdens are often assumed by the other partner (henceforth referred to as the caregiver-partner), which can have negative effects on the caregiver-partner’s well-being and the relationship quality of the dyad.^7–15^ However, as kidney transplantation (KT) has been shown to decrease burden among caregivers in general compared with dialysis, caregiver burden and its related effects may also be modifiable among partners of ESRD patients.^16,17^

Caregiver-partners often come forward as potential living kidney donors for their loved ones who are on dialysis (henceforth referred to as patient-partners). In 2018, over 6400 living kidney donations were performed in the United States, 12% of which were donated by spouses and partners.^18^ It is likely that many more caregiver-partners were willing to donate^19^ but were declined because of a perceived unacceptable risk profile.^20–22^ However, these caregiver-partners likely share households and caregiving responsibilities for the patient-partner, such that the donor’s and the recipient’s health and well-being are interdependent,^23^ and they may experience tangible benefits in terms of caregiver burden, QOL, and relationship quality when the patient-partner receives a transplant. These benefits could be considered by transplant hospitals’ donor selection committees when evaluating interdependent donor candidates, but an empirically derived framework for this does not yet exist.

We hypothesized that caregiver-partners, in interdependent relationships with the patient-partner, would experience changes in QOL, caregiver burden, and relationship quality throughout the patient-partner’s treatment progression. To quantify the potential benefits of living donation between dyads, we studied changes in these areas associated with 2 transitions: (1) when the patient-partner initiates dialysis and (2) when the patient-partner receives a KT.

## MATERIALS AND METHODS

### Study Population and Recruitment

Spouses and partners of dialysis patients and KT recipients at our center were eligible for this study if they spoke English, shared a household with the patient-partner, and if the patient-partner had been on dialysis for at least 6 mo before their transplant or at the time of study participation. Caregiver-partners were recruited at 1 of 3 time-points: pre-KT (in person while attending the patient-partner’s evaluation appointment), at KT (in person while the patient-partner was admitted for KT), or post-KT (in person while attending the patient-partner’s follow-up appointment between 6 mo and 3 y after KT). A single individual could be surveyed multiple times; of 86 individuals who were surveyed at least once, 7 were surveyed twice and 3 were surveyed thrice. If the potential participants were not present in person, they were recruited over the phone with the permission of the patient-partner, or they were referred to the study by the patient-partner. Participants provided written or oral informed consent. This study was approved by the Johns Hopkins University School of Medicine IRB00084611.

### Survey Design

Six validated instruments, commonly used in caregiving, dialysis, and transplantation research, were selected to measure caregiver-partners’ current QOL, caregiver burden, relationship quality, and mental health.^24–29^ QOL was measured using the SF-12, a measure of overall mental and physical health. The SF-12 is widely used in dialysis and transplantation research^30–33^ and has been used among caregivers of dialysis and transplant patients.^34–38^ It has been validated among the general US population as well as among African Americans and dialysis patients specifically.^39–41^ The kidney disease quality of life index consists of the SF-12 and uses several additional kidney-specific questions to capture the effect of kidney disease on QOL. For this study, these additional kidney-specific questions were adapted to capture the effect of the patient-partner’s kidney disease on the caregiver-partner’s QOL; for example, the statement “My kidney disease interferes too much with my life” was adapted to “My partner’s kidney disease interferes too much with my life.” Caregiver burden was measured using the Zarit Caregiving Burden scale, a widely used measure in caregiver research, including among caregivers of ESRD, transplant, and dialysis patients, and validated in a North American population.^16,17,24,42–50^ Mental health was measured using the Patient Health Questionnaire-2, which is widely used to screen for depression among caregivers and has been validated among adults in the United States.^28,51–55^ Relationship satisfaction was measured using the Satisfaction with Married Life scale, and relationship strain was measured using the Revised Dyadic Adjustment Scale, which have both been used among caregivers and validated among couples living in the United States.^25,27,56–62^ In addition, the patient-partner’s comorbidities were assessed with the Charlson Comorbidity Index.

The survey instrument was developed with input from a transplant surgeon and statistician and was pilot tested with 6 caregiver-partners (Appendix 1, SDC, http://links.lww.com/TXD/A252), 2 of whom were recruited at KT evaluation, 1 of whom was recruited at KT admission, and 3 of whom were recruited within 3 y of KT. Pilot participants were given the validated instruments and participated in a semistructured interview to elicit any themes not captured in the validated tools.

While pilot testing the survey, several themes emerged as particularly important to the experience of caregiver-partners of dialysis and KT patients: time, stress, social life, and sexual relations. To compare changes in these specific aspects of caregiver burden and relationship quality over the course of dialysis initiation and transplantation, several individual survey items were adapted from the validated tools to capture the outcomes of interest at multiple time-points. Caregiver-partners were asked to answer these questions for both their current time-point and for their previous time-point; if a caregiver-partner was recruited pre-KT, they were asked both about their current status and about their status before the patient-partner initiated dialysis (predialysis). Likewise, if a caregiver-partner was recruited post-KT, they were asked several questions about their current status and several analogous questions about their status, while the patient-partner was on dialysis (Appendix 1, SDC, http://links.lww.com/TXD/A252).

### Survey Administration

The finalized surveys were administered over the phone or online to caregiver-partners at 3 time-points: pre-KT, at KT, and post-KT from August 2016 to March 2019. Participants who were recruited at KT were given the same survey as those who were recruited pre-KT. Participants who were recruited pre KT or at KT were also eligible to complete a post-KT survey 6 mo post-KT. Participants were given a $10 retail gift card for their participation.

### Statistical Analysis

Validated measures were scored using standard approaches. The SF-12 was scored using a raw scores method with combined mental and physical scores; a higher score indicates better mental and physical health.^63^ SF-12 scores were treated as continuous. Zarit Burden Scale scores were also treated as a continuous variable; possible scores range from 0 to 88, and higher scores indicate higher levels of burden. The Revised Dyadic Adjustment Scale was converted to a binary categorical variable (distressed and nondistressed) using a previously defined cutoff score of 48.^60^ The Satisfaction with Married Life Scale was converted to a binary categorical variable (low satisfaction and high satisfaction) using the mean scores of a nationally representative sample.^27^ The Patient Health Questionnaire-2 was also converted to a binary categorical variable (negative or positive screen for depression) using a previously defined cutoff score of 3.^51^

All analyses were performed using Stata 14.0/MP for Linux (College Station, Texas). Survey items with Likert-type scales were dichotomized for ease of interpretation. Relationships between the patient’s dialysis/KT status and the caregiver-partner’s caregiver burden, QOL, relationship quality, and mental health were assessed using Fisher’s exact tests and Chi-square tests for categorical variables and Wilcoxon-Mann-Whitney tests for continuous variables.

### Sensitivity Analysis

Responses from participants who completed >1 survey were treated as separate observations. To determine if responses from participants who completed >1 survey biased our findings, we performed a sensitivity analysis including only the post-KT survey of those who completed >1. When only the post-KT surveys of those who completed >1 were included, we had a total of 93 survey responses. Inferences from the sensitivity analysis did not change.

## RESULTS

### Study Population

**FIGURE 1.**
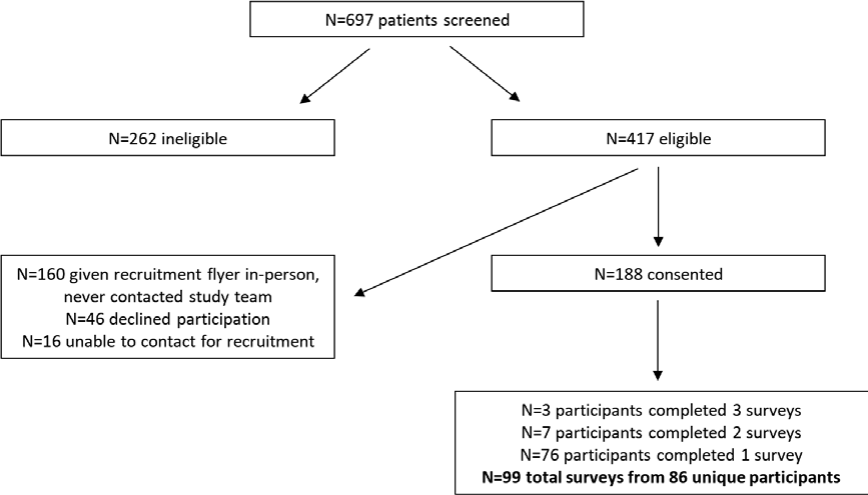
Study recruitment.

Among 697 dialysis patients and KT recipients screened for this study (Figure 1), 262 were ineligible (on dialysis ≤6 mo, not in a cohabiting relationship, caregiver-partner was not English speaking). Among the 417 patients with eligible caregiver-partners, 188 caregiver-partners consented, 168 were attempted to be contacted by phone or email, and 86 participated in the study. After initial survey distribution, the number of pretransplant participants outnumbered those posttransplant, so we ceased to contact or recruit more pretransplant participants. Three participants completed 3 surveys (pre-KT, at KT, and post-KT) and 7 participants completed 2 surveys (pre-KT and post-KT) resulting in 99 total surveys administered.

Among caregiver-partners who participated in the study, the median age was 59 y (interquartile range [IQR], 49–66), 55.1% were white/Caucasian, 77.1% were woman, and 55.3% had greater than a high school degree (Table 1). Caregiver-partners reported that patient-partners had been on dialysis for a median of 1.4 y (IQR, 0.9–2.3) and had a median of 2 comorbidities; 49% reported that patient-partner’s overall health was “fair” or “poor.” Among those patient-partners who had received a transplant, 9 had received a living donor organ, 2 of which were given by the caregiver-partner, or on their behalf in a paired exchange.

**TABLE 1.**
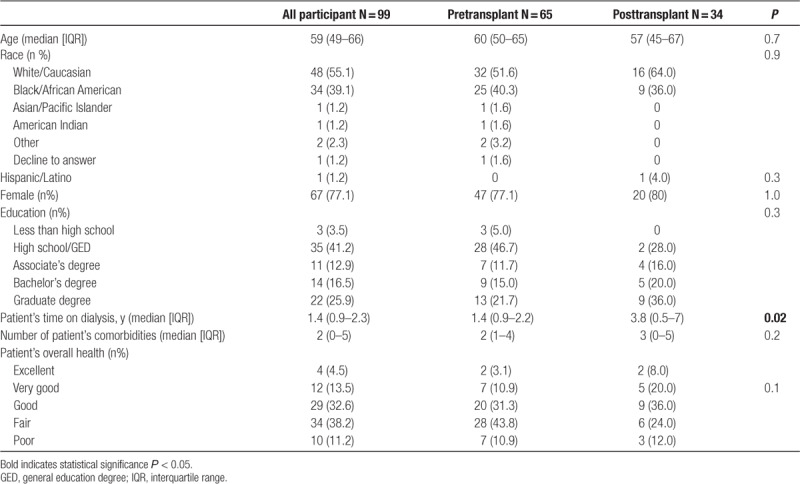
Characteristics of the study population

### Overall QOL, Caregiver Burden, Relationship Quality, and Mental Health

Caregiver-partners who were surveyed after their patient-partners underwent KT had higher SF-12 scores, indicating better QOL, compared with those who were surveyed before their patient-partners underwent KT (*P* = 0.03; Table 2). Among all caregiver-partners, 81.8% were in relationships found to be nondistressed, but 41.4% indicated low relationship satisfaction. However, there was no evidence of differences in overall relationship strain (80.0% versus 85.3% nondistressed; *P* = 0.6) or relationship satisfaction (43.1% versus 38.2% low satisfaction; *P* = 0.6) between pre-KT and post-KT responses. The median caregiving score on the Zarit Caregiving scale was 18.5 (IQR, 11–32) indicating an overall moderate caregiver burden; however, there was no evidence of differences in overall caregiver burden between pre-KT and post-KT responses (median score = 19, pre-KT; median score = 16, post-KT; *P* = 0.2). Of caregiver-partners, 95.4% screened negative for depression; again, there was no evidence of differences in depression between pre-KT and post-KT responses (94.6% negative screen pre-KT; 96.7% negative screen post-KT; *P* = 1.0).

**TABLE 2.**
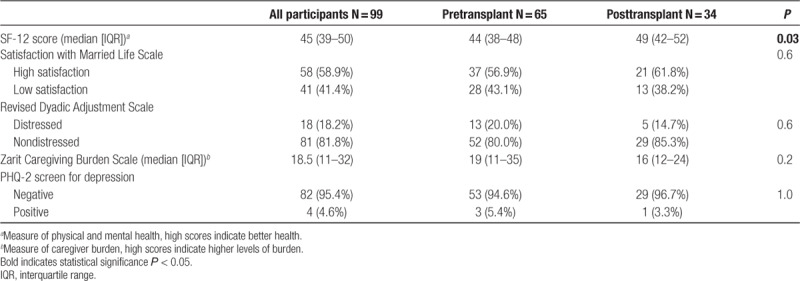
Overall quality of life, relationship quality, caregiver burden, and mental health among caregiver-partners of pre and post kidney transplant patients

### Specific Measures of Caregiver Burden and Relationship Quality

#### Negative Changes Upon Dialysis Initiation

Caregiver-partners reported several negative changes in caregiver burden and relationship quality after their patient-partner initiated dialysis (Table 3). In terms of overall caregiver burden, caregiver-partners were more likely to report being more than a little burdened after the patient-partner initiated dialysis (20.9% at least moderately burdened predialysis versus 29.6% on-dialysis; *P* = 0.03). Likewise, caregiver-partners reported that they were more likely to feel that their social life suffered because of caring for the patient-partner (44.1% at least sometimes felt their social life suffered predialysis versus 44.8% on dialysis; *P* = 0.02), and more likely to feel stressed between caring for the patient-partner and trying to meet other responsibilities (51.7% at least sometimes felt stressed predialysis versus 55.2% on dialysis; *P* < 0.01) after dialysis initiation. However, caregiver-partners were more likely to feel as if they did not have enough time for themselves because of the time spent with the patient-partner *before* dialysis initiation, as compared with after dialysis initiation (41.4% at least sometimes felt they did not have enough time predialysis versus 37.5% on dialysis; *P* < 0.01).

**TABLE 3.**
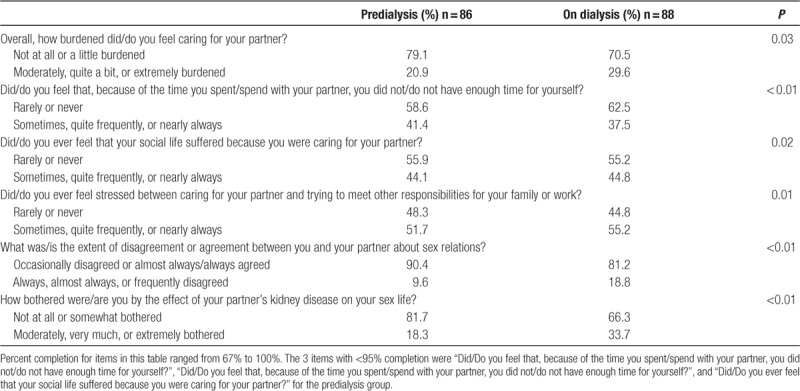
Changes in specific aspects of caregiver-partner caregiver burden and relationship quality upon patient-partner dialysis initiation

Caregiver-partners also reported negative changes in their sexual relationship with the patient-partner: after their patient-partner’s dialysis initiation, caregiver-partners were more likely to report at least frequent disagreement about sex relations (9.6% at least frequent disagreement versus predialysis 18.8% on dialysis; *P* < 0.01) and were more likely to be at least moderately bothered by the effect of the patient-partner’s kidney disease on their sex life (18.3% at least moderately bothered predialysis versus 33.7% on dialysis; *P* < 0.01).

#### Positive Changes With Kidney Transplantation

Caregiver-partners reported several positive changes in caregiver burden and relationship quality after the patient-partner underwent KT (Table 4). In terms of overall caregiver burden, caregiver-partners were less likely to report being more than a little burdened after the patient-partner received a KT (29.6% at least moderately burdened on dialysis versus 16.7% post-KT; *P* = 0.03). Caregiver-partners also reported that, after their patient-partner’s KT, they were less likely to feel they did not have time for themselves because of time spent with the patient-partner (37.5% at least sometimes felt they did not have enough time on dialysis versus 16.7% post-KT; *P* < 0.01), less likely to feel that their social life suffered because of caring for the patient-partner (44.8% at least sometimes felt their social life suffered on dialysis versus 23.3% post-KT; *P* < 0.01), and less likely to feel stressed between caring for the patient-partner and trying to meet other responsibilities (55.2% at least sometimes stressed on dialysis versus 33.3% post-KT; *P* = 0.02). However, there was no evidence of differences in the hours per day caregiver-partners reported spending helping their patient-partner with health-related tasks (*P* = 0.2).

**TABLE 4.**
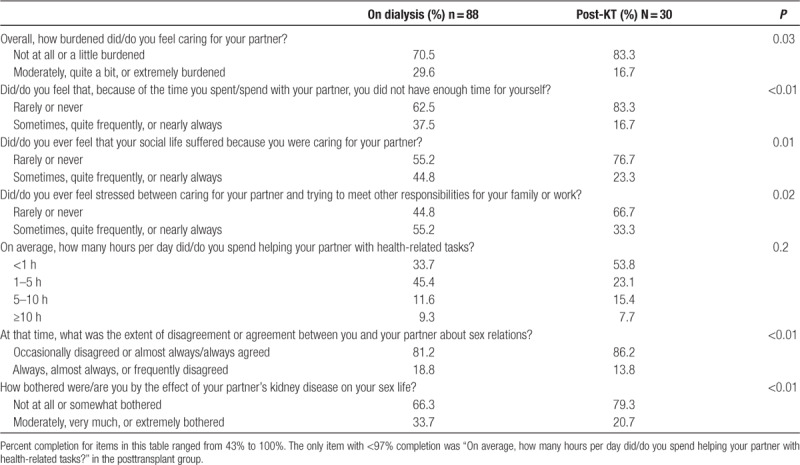
Changes in specific aspects of caregiver-partner caregiver burden and relationship quality after patient-partner kidney transplantation

Caregiver-partners also reported positive changes in sexual relations with their patient-partner; after dialysis initiation caregivers were less likely to report at least frequent disagreement about sexual relations (18.8% at least frequent disagreement on dialysis versus 13.8% post-KT; *P* < 0.01) and were less likely to be at least moderately bothered by the effect of their patient-partner’s kidney disease on their sex life (18.8% at least moderately bothered on dialysis versus 20.7% post-KT; *P* < 0.01).

#### Return to Predialysis Burden After Kidney Transplantation

Improvements in caregiving burden and sexual relationships were of sufficient impact that caregiver-partners returned to predialysis levels after their patient-partner’s KT. Levels of caregiver burden before their patient-partner’s dialysis initiation and after KT were similar (20.9% at least moderately burdened predialysis versus 16.7% post-KT; *P* = 0.5), as were levels of disagreement about sexual relations (9.6% at least frequent disagreement predialysis versus 13.8% post-KT; *P* = 0.1). Likewise, the degree to which they were bothered by the effect of their patient-partner’s kidney disease on their sex life returned to predialysis levels after KT (18.3% at least moderately bothered predialysis versus 20.7% post-KT; *P* = 0.6).

## DISCUSSION

In this study of caregiver-partners (spouses and partners of dialysis patients and KT recipients), participants experienced improvements in overall QOL after their patient-partner transitioned from dialysis to KT. Caregiver-partners also experienced increases in specific aspects of caregiver burden and changes to their sexual relationship after the patient-partners initiated dialysis, but benefits in both areas after the patient-partner received a KT. The magnitude of improvement associated with KT was such that specific aspects of caregiver burden and relationship strain returned to predialysis levels after KT.

Our findings are consistent with a systematic review of quantitative studies of caregiving burden and QOL among caregivers to dialysis patients, which found that among 61 studies of 5387 caregivers, caregiver burden was higher and QOL was lower among dialysis patient caregivers compared with the general population.^5^ Furthermore, 2 cross-sectional surveys from Turkey comparing hemodialysis (n = 133) and peritoneal (n = 113) patient caregivers with KT recipient caregivers found that post-KT caregivers were less burdened than dialysis caregivers.^16,17^ Our study also found improvements in caregiver-partners’ overall QOL when patient-partners underwent KT, as well as improvements in specific aspects of caregiver burden. A single-center cross-sectional survey of 79 partner-caregivers of transplant candidates and recipients found lower levels of life satisfaction among pre-KT responses compared with post-KT responses, but found no significant differences in QOL or caregiving strain.^64^ Our study adds to this literature by assessing changes in caregiver burden and relationship quality before dialysis, after dialysis, and post-KT among caregiver-partners who are in interdependent relationships with the patient. Of note, a multicenter study of 193 living kidney donors in the United States found that, although rare, some donors do experience adverse psychosocial outcomes such as body image concerns and anxiety regarding their remaining kidney function.^65^ These potential risks should also be addressed during donor evaluations.

Our findings are also consistent with prior studies on caregiver burden in other chronic illnesses. A literature review of 25 quantitative and qualitative studies of partner-caregivers of cancer patients found they experienced limited social support, limited social interaction, and insufficient time to meet conflicting responsibilities.^66^ Likewise, caregiver-partners in our study reported negative changes in their social life, increased stress meeting responsibilities, and insufficient time for themselves. Furthermore, a literature review of 78 quantitative and qualitative studies of stroke in working-age adults found deterioration in sexual relations in dyads after stroke in 1 partner,^12^ an experience also reported by caregiver-partners in our study after the patient-partner initated dialysis. Interestingly, although caregiver-partners in our study felt they had more time for themselves after KT compared with when the patient-partner was on dialysis, caregiver-partners also felt they had more time for themselves after dialysis initiation, compared with before dialysis initiation.

This study has several limitations. First, the single-center, English-speaking sample limits its generalizability; however, the study population was heterogeneous in terms of race, sex, and education level. Second, the relatively small sample size limited our ability to detect independent associations between the overall validated instruments and transplant status. Despite this, the items asking caregiver-partners to report specific aspects of caregiver burden and relationship quality at multiple time-points allowed us to assess perceived changes in these factors. Although these items may be subject to more recall bias than items asking about current status, it could be argued that perceived changes in caregiver burden and relationship quality are equally important as measurements actually taken at the time-points of interest. Third, the participation of 86 participants out of 188 consented and 417 eligible is low and suggests that selection bias may be an important limitation. Fourth, we were unable to determine whether or not any caregiver-partners were evaluated and denied for living donation, despite the likely unique experiences of these caregivers.^19,67^ Finally, we were also unable to compare changes in perceived benefit over time since the patient-partner’s transplant. Future work should explore these areas to more fully capture the experiences of caregiver-partners.

Caregiver-partners experienced negative changes in caregiver burden and relationship quality while patient-partners are on dialysis, and subsequent improvements in specific aspects of caregiver burden and relationship quality following the patient-partner’s KT. Specifically, caregiver-partners reported benefits in personal time, social life, stress, sexual relations, and overall QOL after their partner received a transplant. These improvements were of sufficient impact that caregiver-partners reported similar levels of caregiver burden after their partner underwent KT as before their partner ever initiated dialysis. These findings highlight the importance of preemptive transplantation as a means of reducing caregiver burden, as well as the need for more substantial caregiver support. Furthermore, we have previously suggested that the living donor screening and evaluation process should consider benefits to donors, particularly for dyadic donors whose well-being is interdependent with the recipient.^23^ The benefits identified in this study could be among those considered as part of a risk-assessment framework for interdependent donors. The inclusion of benefits would allow for a more comprehensive consideration of the donor’s well-being during the evaluation process. Furthermore, including benefits in a balanced risk-benefit framework of living kidney donation may also allow some donor candidates with risk profiles slightly exceeding existing center thresholds to proceed with donation.

## Supplementary Material


