# Supplementary material for: Kidney Dyads: Caregiver Burden and Relationship Strain Among Partners of Dialysis and Transplant Patients
Source: Transplant Direct. 2020 Jun 8;6(7):e566. doi: 10.1097/TXD.0000000000000998 (PMC7339348; doi:10.1097/TXD.0000000000000998)
Supplement: Supplementary file 1 [file txd-6-e566-s001.pdf]

Appendix 1

QUESTIONS ASKED OF ALL PARTICIPANTS

DEMOGRAPHICS/BACKGROUND

The following questions are about you.
What is your age? ___________
What is your zip code? ___________
Which race best describes you (mark all that apply)

- White/Caucasian
- Asian/Pacific Islander
- Black/African American
- American Indian
- Other
- Decline to Answer

Do you consider yourself Hispanic or Latino?

- Yes
- No

What is the highest level of education that you have completed?

- Less than high school
- High school or GED
- Associate's degree
- Bachelor's degree
- Graduate degree
- Decline to answer

The following questions are about your partner.
Which race best describes your partner (mark all that apply)

- White/Caucasian
- Asian/Pacific Islander
- Black/African American
- American Indian
- Other
- Decline to Answer

Does your partner consider him/herself to be Hispanic or Latino?

- Yes
- No

What is the highest level of education that your partner has completed?

- Less than high school
- High school or GED
- Associate's degree
- Bachelor's degree

- Graduate degree
- Decline to answer

The following questions are about your partner's health

Has your partner ever had a transplant of any organ

- Yes
- No

[If yes] How many transplants has your partner had?

- 1
- 2
- 3
- 4
- 5

[Ask for each transplant] For which organ was your partner's first/second/third/fourth/fifth transplant? (select all that apply)

- Kidney
- Liver
- Pancreas
- Other

How would you describe your partner's health currently?

- Excellent
- Very good
- Good
- Fair
- Poor

The following questions are about both you and your partner

Which best describes your relationship to your partner?

- Married
- Civil union
- Domestic partner
- Co-habiting partner
- Other

How long have you and your partner been in a relationship (please state in years) _____

Do you or your partner have children?

- Yes
- No

How many children do you and your partner have

- 1
- 2
- 3
- 4
- 5
- 6
- 7

- 8
- 9
- 10
- More than 10

How many of your children are under 13 years old? ______

Does anyone else live with you and your partner?

- Yes
- No

Who else do you live with? Mark all that apply.

- Children under age 18
- Children over age 18
- My parents
- My partner's parents
- Live-in paid staff, assistants, caregivers, etc.
- Other
    - If "other," who else lives with you and your partner? ______________________

SF-12

For the following questions, please choose the answers that best describes your experience

In general, would you say your health is

- Excellent
- Very good
- Good
- Fair
- Poor

The following items are about activities you might do during a typical day. Does your health now limit you in these activities? If so, how much?

Moderate activities such as moving a table, pushing a vacuum cleaner, bowling, or playing golf

- Yes, limited a lot
- Yes, limited a little
- No, not limited at all

Climbing several flights of stairs

- Yes, limited a lot
- Yes, limited a little
- No, not limited at all

During the past 4 weeks how much did pain interfere with your normal work (including both work outside the home and housework)?

- Extremely
- Quite a bit
- Moderately
- A little bit
- Not at all

During the past 4 weeks have you had any of the following problems with your work or other regular daily activities as a result of your physical health?

Accomplished less than you would like

- All of the time
- Most of the time
- Some of the time
- A little of the time
- None of the time

Were limited in the kind of work or other activities

- All of the time
- Most of the time
- Some of the time
- A little of the time
- None of the time

During the past 4 weeks have you had any of the following problems with your work or other regular daily activities as a result of any emotional problems (such as feeling depressed or anxious)?

Accomplished less than you would like

- All of the time
- Most of the time
- Some of the time
- A little of the time
- None of the time

Did work or other activities less carefully than usual

- All of the time
- Most of the time
- Some of the time
- A little of the time
- None of the time

These questions are about how you feel and how things have been with you during the past 4 weeks. For each question, please give the one answer that comes closest to the way you have been feeling. How much of the time during the past 4 weeks:

Have you felt calm and peaceful?

- All of the time
- Most of the time
- Some of the time
- A little of the time
- None of the time

Did you have a lot of energy?

- All of the time
- Most of the time
- Some of the time
- A little of the time
- None of the time

Have you felt downhearted and blue?

- All of the time
- Most of the time
- Some of the time
- A little of the time
- None of the time

During the past 4 weeks how much of the time has your physical health or emotional problems interfered with your social activities (like visiting with friends, relatives, etc.)?

- All of the time
- Most of the time
- Some of the time
- A little of the time
- None of the time

QUESTIONS ADAPTED FROM THE SF-12

During the past 4 weeks, have you had any of the following problems with your work or other regular daily activities as a result of your partner's physical health?

Accomplished less than you would like

- Yes
- No

Were limited in the kind of work or other activities

- Yes
- No

During the past 4 weeks, have you had any of the following problems with your work or other regular daily activities as a result of any emotional problems (such as feeling depressed or anxious) that your partner has had?

Accomplished less than you would like

- Yes
- No

Did work or other activities less carefully than usual

- Yes
- No

During the past 4 weeks, how much did your partner's pain interfere with your normal work (including both work outside the home and housework)?

- Extremely
- Quite a bit
- Moderately
- A little bit
- Not at all

During the past 4 weeks, how much of the time has your partner's physical health or emotional problems interfered with your social activities (like visiting with friends, relatives, etc.)?

- All of the time

- Most of the time
- Some of the time
- A little of the time
- None of the time

REVISED DYADIC ADJUSTMENT SCALE

Most people have disagreements in their relationships. Please indicate below the extent of agreement or disagreement between you and your partner for each item.

Religious matters

- Always agree
- Almost always agree
- Occasionally disagree
- Frequently disagree
- Almost always disagree
- Always disagree

Demonstrations of affection

- Always agree
- Almost always agree
- Occasionally disagree
- Frequently disagree
- Almost always disagree
- Always disagree

Making major decisions

- Always agree
- Almost always agree
- Occasionally disagree
- Frequently disagree
- Almost always disagree
- Always disagree

Sex relations

- Always agree
- Almost always agree
- Occasionally disagree
- Frequently disagree
- Almost always disagree
- Always disagree

Conventionality, correct or proper behavior

- Always agree
- Almost always agree
- Occasionally disagree
- Frequently disagree
- Almost always disagree
- Always disagree

Career decisions

- Always agree
- Almost always agree
- Occasionally disagree

- Frequently disagree
- Almost always disagree
- Always disagree

The following questions are of personal nature. Remember you may decline to answer

How often do you discuss or have you considered divorce, separation, or terminating your relationship?

- All of the time
- Most of the time
- More often than not
- Occasionally
- Rarely
- Never

Do you ever regret that you married or lived together?

- All of the time
- Most of the time
- More often than not
- Occasionally
- Rarely
- Never

How often do you and your partner quarrel?

- All of the time
- Most of the time
- More often than not
- Occasionally
- Rarely
- Never

How often do you and your partner "get on each other's nerves?"

- All of the time
- Most of the time
- More often than not
- Occasionally
- Rarely
- Never

Do you and your partner engage in outside interests together?

- All of the time
- Most of the time
- More often than not
- Occasionally
- Rarely
- Never

How often would you say the following events occur between you and your partner?

Have a stimulating exchange of ideas?

- Never
- Less than once a month

- Once or twice a month
- Once or twice a week
- Once a day
- More often

Calmly discuss something

- Never
- Less than once a month
- Once or twice a month
- Once or twice a week
- Once a day
- More often

Work together on a project

- Never
- Less than once a month
- Once or twice a month
- Once or twice a week
- Once a day
- More often

Please choose the answer that best describes the degree of happiness, all things considered, of your relationship. The middle point, "happy," represents the degree of happiness of most relationships.

- Extremely unhappy
- Fairly unhappy
- A little unhappy
- Happy
- Very happy
- Extremely happy
- Perfect

QUESTIONS ADAPTED FROM THE REVISED DYADIC ADJUSTMENT SCALE

Most people have disagreements in their relationships. Please indicate below the extent of agreement or disagreement between you and your partner for each item.

Handling family finances

- Always agree
- Almost always agree
- Occasionally disagree
- Frequently disagree
- Almost always disagree
- Always disagree

Matters of recreation

- Always agree
- Almost always agree
- Occasionally disagree

- Frequently disagree
- Almost always disagree
- Always disagree

Household tasks

- Always agree
- Almost always agree
- Occasionally disagree
- Frequently disagree
- Almost always disagree
- Always disagree

SATISFACTION WITH MARRIED LIFE SCALE

Below are five statements with which you may agree or disagree. Indicate your agreement with each item. Please be open an honest in responding.

In most ways my married life or relationship is close to ideal

- Strongly disagree
- Disagree
- Slightly disagree
- Neither agree nor disagree
- Slightly agree
- Agree
- Strongly agree

The conditions of my married life or relationship are excellent

- Strongly disagree
- Disagree
- Slightly disagree
- Neither agree nor disagree
- Slightly agree
- Agree
- Strongly agree

I am satisfied with my married life or relationship

- Strongly disagree
- Disagree
- Slightly disagree
- Neither agree nor disagree
- Slightly agree
- Agree
- Strongly agree

So far I have gotten the important things I want in my married life or relationship

- Strongly disagree
- Disagree
- Slightly disagree

- Neither agree nor disagree
- Slightly agree
- Agree
- Strongly agree

If I could live my married life or relationship over, I would change almost nothing

- Strongly disagree
- Disagree
- Slightly disagree
- Neither agree nor disagree
- Slightly agree
- Agree
- Strongly agree

QUESTIONS ADAPTED FROM THE KIDNEY DISEASE QUALITY OF LIFE INDEX

How true or false is each of the following statements for you?

My partner's kidney disease interferes too much with my life

- Definitely true
- Mostly true
- Don't know
- Mostly false
- Definitely false

Too much of my time is spent dealing with my partner's kidney disease

- Definitely true
- Mostly true
- Don't know
- Mostly false
- Definitely false

I feel frustrated dealing with my partner's kidney disease

- Definitely true
- Mostly true
- Don't know
- Mostly false
- Definitely false

I feel like my partner is a burden on my family

- Definitely true
- Mostly true
- Don't know
- Mostly false
- Definitely false

Some people are bothered by the effects of kidney disease on their daily life, while others are not. How much does your partner's kidney disease bother you in each of the following areas?

Fluid retention (holding onto excess fluid)

- Not at all bothered
- Somewhat bothered
- Moderately bothered
- Very much bothered
- Extremely bothered

Dietary restrictions

- Not at all bothered
- Somewhat bothered
- Moderately bothered
- Very much bothered
- Extremely bothered

Your partner's ability to work around the house

- Not at all bothered
- Somewhat bothered
- Moderately bothered
- Very much bothered
- Extremely bothered

Your ability to travel

- Not at all bothered
- Somewhat bothered
- Moderately bothered
- Very much bothered
- Extremely bothered

Being dependent on doctors and other medical staff

- Not at all bothered
- Somewhat bothered
- Moderately bothered
- Very much bothered
- Extremely bothered

Stress or worries caused by kidney disease

- Not at all bothered
- Somewhat bothered
- Moderately bothered
- Very much bothered
- Extremely bothered

Your sex life

- Not at all bothered
- Somewhat bothered
- Moderately bothered
- Very much bothered
- Extremely bothered

Your personal appearance

- Not at all bothered

- Somewhat bothered
- Moderately bothered
- Very much bothered
- Extremely bothered

Your partner’s personal appearance

- Not at all bothered
- Somewhat bothered
- Moderately bothered
- Very much bothered
- Extremely bothered

ZARIT CAREGIVING BURDEN SCALE

The following is a list of statements which reflect how people sometimes feel when taking care of another person. After each statement, indicate how often you feel that way: never, rarely, sometimes, quite frequently, or nearly always. There are no right or wrong answers.

Do you feel that your partner asks for more help than he or she needs?

- Never
- Rarely
- Sometimes
- Quite Frequently
- Nearly always

Do you feel that, because of the time you spend with your partner, you don't have enough time for yourself?

- Never
- Rarely
- Sometimes
- Quite Frequently
- Nearly always

Do you feel stressed between caring for your partner and trying to meet other responsibilities for your family or work?

- Never
- Rarely
- Sometimes
- Quite Frequently
- Nearly always

Do you feel embarrassed about your partner’s behavior?

- Never
- Rarely
- Sometimes
- Quite Frequently
- Nearly always

Do you feel angry when you are around our partner?

- Never

- Rarely
- Sometimes
- Quite Frequently
- Nearly always

Do you feel that your partner currently affects your relationship with other family members?

- Never
- Rarely
- Sometimes
- Quite Frequently
- Nearly always

Are you afraid about what the future holds for your partner?

- Never
- Rarely
- Sometimes
- Quite Frequently
- Nearly always

Do you feel that your partner is dependent upon you?

- Never
- Rarely
- Sometimes
- Quite Frequently
- Nearly always

Do you feel strained when you are around your partner?

- Never
- Rarely
- Sometimes
- Quite Frequently
- Nearly always

Do you feel that your health has suffered because of your involvement with your partner?

- Never
- Rarely
- Sometimes
- Quite Frequently
- Nearly always

Do you feel that you don’t have as much privacy as you would like, because of your partner?

- Never
- Rarely
- Sometimes
- Quite Frequently
- Nearly always

Do you feel that your social life has suffered because you are caring for your partner?

- Never
- Rarely

- Sometimes
- Quite Frequently
- Nearly always

Do you feel uncomfortable having your friends over because of your partner?

- Never
- Rarely
- Sometimes
- Quite Frequently
- Nearly always

Do you feel that your partner seems to expect you to take care of him or her, as if you were the only one he or she could depend on?

- Never
- Rarely
- Sometimes
- Quite Frequently
- Nearly always

Do you feel that you don't have enough money to care for your partner, in addition to the rest of your expenses?

- Never
- Rarely
- Sometimes
- Quite Frequently
- Nearly always

Do you feel that you will be unable to take care of your partner much longer?

- Never
- Rarely
- Sometimes
- Quite Frequently
- Nearly always

Do you feel that you have lost control of your life since your partner's illness?

- Never
- Rarely
- Sometimes
- Quite Frequently
- Nearly always

Do you wish that you could just leave the care of your partner to someone else?

- Never
- Rarely
- Sometimes
- Quite Frequently
- Nearly always

Do you feel uncertain about what to do about your partner?

- Never

- Rarely
- Sometime
- Quite Frequently
- Nearly always

Do you feel that you should be doing more for your partner?

- Never
- Rarely
- Sometimes
- Quite Frequently
- Nearly always

Do you feel that you could do a better job in caring for your partner?

- Never
- Rarely
- Sometimes
- Quite Frequently
- Nearly always

Overall, how burdened do you feel in caring for your partner?

- Not at all
- A little
- Moderately
- Quite a bit
- Extremely

PATIENT HEALTH QUESTIONNAIRE 2

Over the last 2 weeks, how often have you been bothered by any of the following problems?
Little interest or pleasure in doing things

- Not at all
- Several days
- More than half the days
- Nearly every day

Feeling down, depressed, or hopeless

- Not at all
- A little
- Moderately
- Quite a bit
- Extremely

CHARLSON COMORBIDITY INDEX

The next set of questions will list some health conditions. Please say if a physician or healthcare provider has diagnosed your partner with any of these conditions. You may say yes, no, or don't know.
Myocardial infarction or heart attack

- Yes

- No
- Don’t know

Peripheral vascular disease such as intermittent claudication, acute arterial insufficiency, or thoracic or abdominal aneurysm

- Yes
- No
- Don’t know

Cerebral vascular disease such as stroke or transient ischemic attack (mini stroke)

- Yes
- No
- Don’t know

Dementia

- Yes
- No
- Don’t know

Chronic lung disease

- Yes
- No
- Don’t know

Rheumatological disease, such as lupus, connective tissue disease or rheumatoid arthritis

- Yes
- No
- Don’t know

Peptic ulcer disease

- Yes
- No
- Don’t know

Diabetes

- Yes
- No
- Don’t know

Diabetes with complications, such as retinopathy, neuropathy, or nephropathy

- Yes
- No
- Don’t know

Metastatic cancer

- Yes
- No
- Don’t know

Leukemia

- Yes
- No
- Don’t know

Lymphoma

- Yes
- No
- Don’t know

HIV

- Yes
- No
- Don’t know

Does your partner have a disability?

- Yes
- No
- Don’t know

  Visual impairment

  - Yes
  - No
  - Don’t know

  Hearing impairment

  - Yes
  - No
  - Don’t know

  Physical disability

  - Yes
  - No
  - Don’t know

  Walking disability

  - Yes
  - No
  - Don’t know

  Other disability

  - Yes
  - No
  - Don’t know

  If yes: list disability(ies)______________________

QUESTIONS ASKED OF PRETRANSPLANT PARTICIPANTS

The next set of questions will ask about the time before your partner began dialysis
Overall, how burdened did you feel caring for your partner before they started dialysis?

- Not at all
- A little
- Moderately
- Quite a bit
- Extremely

Did you feel that, because of the time you spent with your partner, you didn't have enough time for yourself?

- Never
- Rarely
- Sometimes
- Quite Frequently
- Nearly Always

Did you ever feel that your social life suffered because you were caring for your partner?

- Never
- Rarely
- Sometimes
- Quite frequently
- Nearly always

At that time, did you ever feel stressed between caring for your partner and trying to meet other responsibilities for your family or work?

- Never
- Rarely
- Sometimes
- Quite Frequently
- Nearly Always

The next two questions are personal and relate to your sexual activity before your partner started dialysis.
At that time, what was the extent of disagreement or agreement between you and your partner about sex relations?

- Always agreed
- Almost always agreed
- Occasionally disagreed
- Frequently disagreed
- Almost always disagreed
- Always disagreed

At that time, how bothered were you by the effect of your partner's kidney disease on your sex life?

- Always agreed
- Almost always agreed
- Occasionally disagreed

- Frequently disagreed
- Almost always disagreed
- Always disagreed

The next four questions will ask about your life currently, now that your partner is on dialysis

On average, how many hours per day do you spend helping your partner with health related tasks?

- Less than one hour
- 1-3 hours
- 3-5 hours
- 5-7 hours
- 7-10 hours
- 10-12 hours
- more than 12 hours

The following questions will ask about your partner's health

How many months has your partner been on dialysis? _____________

QUESTIONS ASKED OF POSTTRANSPLANT PARTICIPANTS

The next set of questions will ask about the time when your partner was on dialysis, before their transplant.

At that time, on average, how many hours per day did you spend helping your partner with health related tasks?

- Less than one hour
- 1-3 hours
- 3-5 hours
- 5-7 hours
- 7-10 hours
- 10-12 hours
- more than 12 hours

The next few questions are personal and relate to your sexual activity while your partner was on dialysis before their transplant.

At that time, what was the extent of disagreement or agreement between you and your partner about sex relations?

- Always agreed
- Almost always agreed
- Occasionally disagreed
- Frequently disagreed
- Almost always disagreed
- Always disagreed

At that time, how bothered were you by the effect of your partner's kidney disease on your sex life?

- Always agreed
- Almost always agreed
- Occasionally disagreed
- Frequently disagreed
- Almost always disagreed
- Always disagreed

Keep thinking about the time when your partner was on dialysis before the transplant

Overall, how burdened did you feel caring for your partner while they were on dialysis?

- Not at all
- A little
- Moderately
- Quite a bit
- Extremely

Did you feel that, because of the time you spent with your partner, you didn't have enough time for yourself?

- Never
- Rarely
- Sometimes
- Quite Frequently

- Nearly Always

Did you ever feel that your social life suffered because you were caring for your partner?

- Never
- Rarely
- Sometimes
- Quite frequently
- Nearly always

At that time, did you ever feel stressed between caring for your partner and trying to meet other responsibilities for your family or work?

- Never
- Rarely
- Sometimes
- Quite Frequently
- Nearly Always

The next set of questions will ask about life currently, after your partner's transplant.

On average, how many house per day do you spend helping your partner with health related tasks?

- Less than one hour
- 1-3 hours
- 3-5 hours
- 5-7 hours
- 7-10 hours
- 10-12 hours
- more than 12 hours
